# First report of multidrug-resistant and pathogenic *Plesiomonas shigelloides* from endangered crested ibis (*Nipponia nippon*)

**DOI:** 10.1186/s12917-025-04755-3

**Published:** 2025-04-29

**Authors:** Jing Xia, Lele Shao, Xuanyi Chen, Yi Ji, Wulin Ma, Keyuan Chen, Guoqiang Qiu, Houhui Song, Yongchun Yang

**Affiliations:** 1 Key Laboratory of Applied Biotechnology on Animal Science & Veterinary Medicine of Zhejiang Province, Zhejiang Engineering Research Center for Veterinary Diagnostics & Advanced Technology, Zhejiang International Science and Technology Cooperation Base for Veterinary Medicine and Health Management, Belt and Road International Joint Laboratory for One Health and Food Safety, China-Australia Joint Laboratory for Animal Health Big Data Analytics, College of Veterinary Medicine of Zhejiang A&F University, 666 Wusu Street, Lin’an District, Hangzhou, Zhejiang Province 311300 China; 2 Deqing County Ecological Forestry Comprehensive Service Center, Deqing, Zhejiang Province 313200 China

**Keywords:** *Plesiomonas shigelloides*, Crested ibis, Multidrug resistance, Public health

## Abstract

*Plesiomonas shigelloides* (*P. shigelloides*) is a gram-negative, thermotolerant, motile, and pleomorphic microorganism that acts as an opportunistic pathogen, capable of causing abscesses, enteric infections, and even sepsis in humans and other animals, thereby posing a threat to human and animal health worldwide. The crested ibis (*Nipponia nippon*) is a globally endangered avian species with a limited population and is vulnerable for various infections. To gain a better understanding of the prevalence of *P. shigelloides* in the endangered crested ibis, fecal samples from the crested ibis at the Xiazhu Lake Crested Ibis Breeding Research Center, China were collected and analyzed. The results confirmed that 45.6% (36/79) of the fecal samples tested positive for *P. shigelloides*. Antibiotic susceptibility testing showed that 27.8% (10/36) of *P. shigelloides* isolates were multidrug-resistant, with resistance to ampicillin and amikacin being 100% and 77.8%, respectively. Subsequently, ten representative isolates were subjected to whole genome sequencing, and resistance and virulence genes were analyzed. The evolutionary tree of the genomes show that these strains have different origins, with one strain closely related to a strain from human. Finally, the pathogenicity experiment demonstrated that several strains exhibit pathogenicity in both mice and waterfowl, with two strains displaying lethal pathogenicity in mice. To the best of our knowledge, this is the first report of multidrug-resistant and pathogenic *P. shigelloides* isolated from the crested ibis, suggesting a potential threat to this endangered species and broader public health implications.

**Supplementary Information:**

The online version contains supplementary material available at 10.1186/s12917-025-04755-3.

## Introduction

Crested ibis (*Nipponia nippon*) is a flagship species of the endangered wildlife conservation in the world, which is known as an “Oriental gem” in China [1, 2]. Wild populations were almost extinct in the historically widespread areas throughout Eastern Russia, Korea, Japan, to central China. The number of populations decreased sharply due to the deterioration of the environment and other factors [3, 4]. After ornithologists saw the wild crested ibis for the last time in 1964, no one ever discovered them again over a decade. Until 1981, a small, single wild population of crested ibises (consisting of two pairs and three nestlings) was discovered in Yang County, Shaanxi Province, China, by Chinese scientists. Since then, China has initiated and continued the protection of the crested ibis to this very day, making a significant contribution to the maintenance of wildlife biodiversity [4, 5].

*Plesiomonas shigelloides* (*P. shigelloides*) is an opportunistic pathogen that can cause abscesses, enteric infections and even sepsis in humans and other animals, posing a threat to human and animal health, worldwide [6, 7]. *P. shigelloides* is widely distributed and could be isolated from the water environment and various hosts, such as fish, shellfish, crustaceans, waterfowls, mammals, amphibians, reptiles, birds, and other vertebrates [8–11]. Over the past several years, the infection with *P. shigelloides* has attracted increasing attention, particularly in children, the elderly, and those with impaired immune systems. Drinking or even being exposed to untreated water increases the likelihood of suffering from diarrhea caused by *P. shigelloides* [12, 13]. Therefore, it needs to paid attention for the public health brought about by cross-infection of *P. shigelloides*.

Although the population of the crested ibis has significantly increased compared to the past, we still need to maintain ongoing attention to their health. With the expansion of artificial breeding programs, bacterial diseases have become one of the limiting factors in the conservation efforts for the crested ibis. There have been quite a few reports of waterfowls carrying *P. shigelloides* with or without symptoms [14–16]. The scarlet ibis (*Eudocimus ruber*) belongs to the same family of ibises as the crested ibis. In 2016, Castelo-Branco et al. discovered *P. shigelloides* in captive scarlet ibises and their habitats, but no follow-up research was conducted [17]. The overlap between the foraging environment of the crested ibis and the habitat of *P. shigelloides* heightens the risk of infection in these birds.

To better understand the prevalence and evaluate potential risks of *P. shigelloides* in the crested ibis’s population, we chose the only population-gathering site of the crested ibis in southern China, which is located at Xiazhu Lake, Deqing County, Zhejiang Province. Fresh feces of crested ibises were collected. Additionally, we investigated the isolation, molecular characteristics, antibiotic susceptibility, and pathogenicity of the isolated *P. shigelloides* strains. We then assessed the prevalence of *P. shigelloides* in the southern breeding grounds of the crested ibis’s population, analyzed the whole-genome characteristics of representative strains, and evaluated their antibiotic resistance and pathogenicity profiles. We anticipate that these findings will provide a foundation for the prevention of diseases affecting the crested ibis and address the potential public health risks posed by *P. shigelloides.*

## Materials and methods

### Ethical statement

All experiments were conducted in accordance with the guidelines and regulations outlined by the Management and Use of Laboratory Animals of Zhejiang Province and complied with China’s existing laws and regulations for biological research. We only collected feces from the cages of the crested ibises and did not perform any procedures on these birds, minimizing the impact on them.

The animals’ experiments were conducted in accordance with the Regulations for the Administration of Affairs Concerning Experimental Animals approved by the State Council of the People’s Republic of China. The protocol received approval from the Institutional Animal Care and Use Committee of Zhejiang A&F University (Permit Number: ZAFUAC2023001).

### Sample collection

The fresh fecal samples were collected from the crested ibis in Xiazhu Lake Crested Ibis Breeding Research Center, the largest crested ibis breeding base in China, located in Deqing County, Zhejiang Province. At the time of sampling, January 2021, there were 394 of the captive-bred crested ibis, and none of the birds had used antibiotics within 6 months before the study nor showed any evident signs of disease. The crested ibis are known for their timidity and susceptibility to being easily startled. To minimize stress, fecal samples were collected from their enclosures with minimal disturbance. In total, 79 fresh fecal samples were collected with maximum effort. The samples were then transported to the laboratory within 2 h on the day of collection.

### Isolation and biochemical identification of *P. shigelloides*

The bacterial strains were preliminarily identified according to Bergey’s Manual of Systematic Bacteriology and Common Bacterial System Identification Manual. The *P. shigelloides* strains were identified by using bacterial isolation and culture, morphological and biochemical identification. Colonies isolation or purification using brain-heart infusion (BHI), *Salmonella*-*Shigella* (SS) or sheep blood agar plates. In brief, round, colorless, semi-transparent, smooth-surfaced, moist small colonies on SS agar plates of gram-negative bacilli were selected for biochemical identification, including the lactose, sucrose, maltose, raffinose, sorbitol, hydrogen sulfide, lysine decarboxylase, ornithine decarboxylase and amino acid decarboxylase control.

### Sequence analysis of 16S rDNA

The isolates were seeded into BHI broth medium and cultured overnight. Total genomic DNA was isolated from the liquid culture using FastPure Bacteria DNA Isolation Kit (Vazyme) according to the manufacturer’s instructions. The full length of 16S rDNA was amplified by PCR with the universal primers, 27 F (5′-AGAGTTTGATCCTGGCTCAG-3′) and 1492R (5′-GGTTACCTTGTTACGA CTT-3′) [18]. Amplification products were recovered and sequenced by Tsingke Biotechnology (China). The obtained 16S rRNA sequences were analyzed by the NCBI BLAST retrieval system for sequence homology. Then, a phylogenetic tree of the 16S rRNA gene sequences was constructed by comparing the sequences of the isolates with those of other bacterial species retrieved from GenBank [9, 10], using the neighbor-joining method in MEGA software v11.0.10. A bootstrap analysis with 1000 replicates was used to estimate the reliability of each tree topology.

### Molecular typing of *P. shigelloides* by enterobacterial repetitive intergenic consensus (ERIC)-PCR

ERIC-PCR possessed potentially superior resolution merits in intra-typing of *P. shigelloides* [19]. It was performed on different *P. shigelloides* strains with the primers, ERIC-F (5’-ATGTAAGCTCCTGGGGATTCAC-3’) and ERIC-R (5’-AAGTAAG TGACTGGGGTGAGCG-3’). After PCR reaction was performed, the amplification products were placed in 1% agarose gel for electrophoresis and photographed on a UV transilluminator for further analysis by GelJ software v_2_2 refer to previous research [19].

### Antibiotic susceptibility testing

The sensitivity of the isolated strains to antibiotics was investigated using the Kirby–Bauer disc diffusion method, following the procedure detailed by the Clinical and Laboratory Standards Institute (CLSI) [20]. Susceptibility was categorized as susceptible, intermediate or resistant by measuring the diameter of inhibition zone according to the criteria stipulated by the CLSI. *P. shigelloides* strains were subjected to antimicrobial susceptibility to relevant antibiotics including ampicillin, cefotaxime, cefoperazone, meropenem, gentamicin, amikacin, ciprofloxacin, enrofloxacin, norfloxacin, florfenicol, polymyxin B, and tetracycline. *E. coli* ATCC25922 served as quality control.

### Whole-genome sequencing and analysis

Ten representative strains based on results of ERIC-PCR and antibiotics susceptibility testing were selected for the second-generation sequencing, which was commissioned from Institute of Microbiology, Chinese Academy of Sciences. Utilize the CGE website (Center for Genomic Epidemiology) to conduct SNP-based phylogenetic analysis on the whole-genome sequences of ten selected strains of *P. shigelloides*, as well as on thirty-one genomic sequences of *P. shigelloides* isolated from different sources (including human, fish, birds, water, food and unknown) downloaded from the PATRIC database (Table S1). Then, the phylogenomic analysis of the strains was constructed. Meanwhile, the results of whole-genome sequencing were compared with the Virulence Factor Database (VFDB, http://www.mgc.ac.cn/VFs) and the Comprehensive Antibiotic Research Database (CARD, http://arpcard.mcmaster.ca) respectively, for the knowledge of the virulence genes and antimicrobial resistance genes carried by these strains.

### Detection of antibiotic resistance genes

Based on the results of whole genome sequencing, resistance genes, *bla*_TEM_, *aac(6’)-Ib3*, *aac(6’)-Ib-cr*, *mph(A)*, *arr-2*, *tet(A)*, *qacEΔ1*, *dfrA1* and *sulI* were detected by PCR and sequencing for the *P. shigelloides* strains isolated from crested ibis. Primers of the resistance genes referred to the published literatures [21–24].

### Pathogenicity of *P. shigelloides* in mice and ducks

The pathogenicity of ten representative *P. shigelloides* strains was confirmed by conducting experimental challenges on six weeks old ICR mice and one-week-old Muscovy ducks, to evaluate the disease-causing potential of isolated strains both in mammals and waterfowls. Firstly, the strains were grown in BHI broth medium and cultured with agitation at 37℃ for 12 h. The strains were washed twice with 10 mmol/L phosphate-buffered saline (PBS) and then adjusting the optical density (OD) 600 value to 1 × 10^10^ CFU/mL corresponding to the number of bacteria and then enumerating the bacteria with plate counting method. For mice experiment, the ICR mice were separated into PBS control group and bacteria-inoculated groups. The mice were challenged intraperitoneally with 1 × 10^9^ CFU and control group were inoculated intraperitoneally with sterile PBS. Continuous monitoring was conducted on weight loss, clinical symptoms, and behaviors of each mouse for 14 d.p.i. Two strains (*P. shigelloides*-18 and *P. shigelloides* 36) exhibiting significant weight loss were further evaluated for survival rates. Post-mortem examinations were promptly performed on deceased mice to observe pathological changes, and bacterial isolation and identification were conducted from the organs.

For the infection experiment on ducks, one-week-old, clinically healthy Muscovy ducks were used in this study. For each strain, the inoculated groups (three ducks for each group, *n* = 3) were inoculated intraperitoneally with of the indicated bacteria with 1 × 10^9^ CFU. The control group were inoculated intraperitoneally with equal volume of PBS. The birds were also observed for clinical symptoms, morbidity and mortality for up to 14 days.

## Results

### Isolation, identification and typing of *P. shigelloides*

**Fig. 1:**
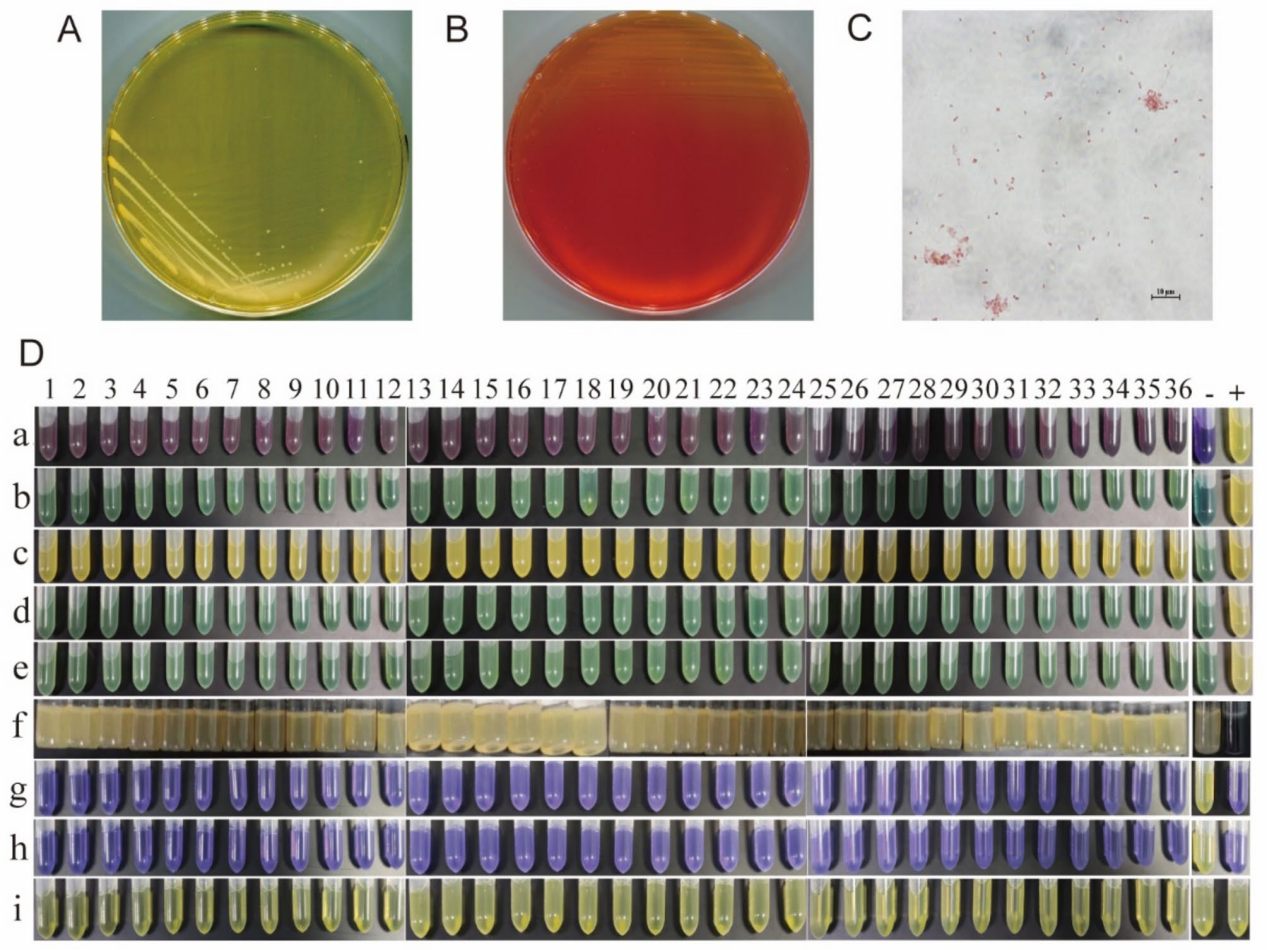
Isolation, identification, and biochemical characteristics of *P. shigelloides*. Colonial morphology of *P. shigelloides* isolate in BHI agar plate (**A**), SS agar plate (**B**) and morphological characteristics (**C**) observed under 100×oil immersion lens with Gram staining. (**D**) Biochemical identification results of all *P. shigelloides* isolates. Biochemical projects a-i refers to lactose, sucrose, maltose, raffinose, sorbitol, hydrogen sulfide, lysine decarboxylase, ornithine decarboxylase and amino acid decarboxylase control

In January 2021, the fecal samples were collected from the crested ibises in Xiazhu Lake crested ibis Breeding Research Center, which is currently the largest crested ibis breeding base in China, located in Deqing County, Zhejiang Province, China. A total of 79 fresh fecal samples were randomly collected from this population. The bacterium grew well on BHI medium, SS medium and sheep blood medium. The colonies were round, beige, opaque, smooth, moist, and slightly raised but with neat edges in BHI medium (Fig. 1A and B). Gram staining results were microscopically observed. The bacterial cells stained red and appeared as short rods, which were arranged singly or in pairs, indicating that the strain was gram-negative (Figure 1C). Meanwhile, biochemical identification results demonstrated that the bacterium could hydrolyze maltose, glucose, and myo-inositol, but not lactose, sucrose, raffinose, or sorbitol. Additionally, positive results were obtained for lysine decarboxylase and ornithine decarboxylase tests (Figure 1D).

**Fig. 2:**
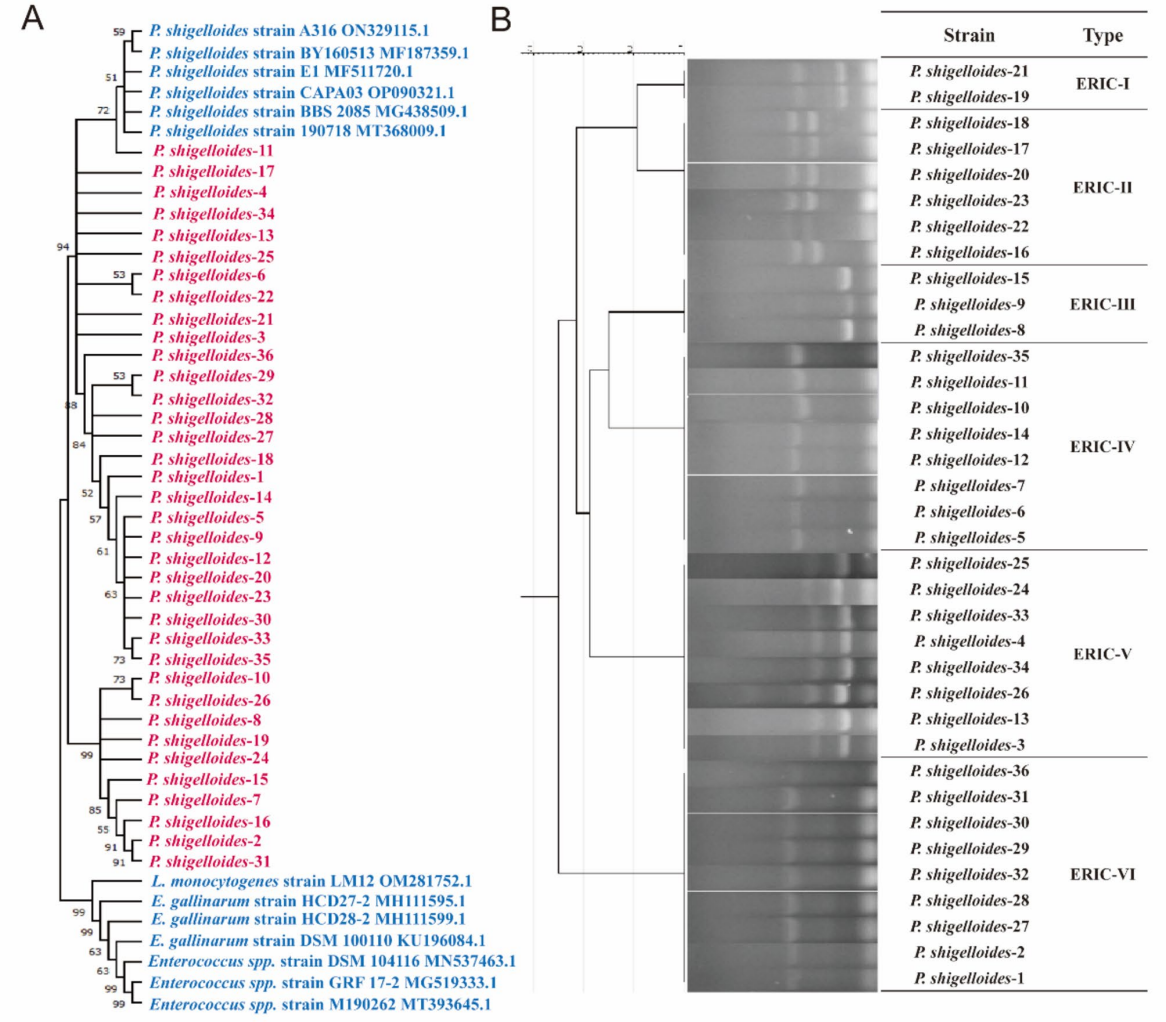
Identification and typing of *P. shigelloides.* (**A**) Phylogenetic tree based on partial 16S rRNA gene sequences and constructed in MEGA software v11.0.10 by using the neighbor-joining method. A bootstrap analysis with 1000 replicates was used to estimate the reliability of each tree topology. (**B**) Molecular typing of *P. shigelloides* by ERIC-PCR

To further characterize the isolates, 16S rRNA genes were amplified and sequenced. Homology analysis using NCBI BLAST revealed a similarity of over 99% between these strains and *P. shigelloides*. Based on 16S rRNA-based phylogeny, these strains were determined to be *P. shigelloides* (Figs. 2A). Therefore, based on the morphological, physiological, biochemical and molecular characteristics, these isolates were identified as *P. shigelloides*. Overall, 45.6% (36/79) of the fecal samples were positive and totally 36 strains of *P. shigelloides* were isolated, these isolates were named as *P. shigelloides* 1–36.

Followed by ERIC-PCR typing, these isolates can be divided into six categories, namely ERIC-I to ERIC-VI types (Fig. 2B). Among them, ERIC-VI type is the most prevalent, containing nine isolates. Followed by ERIC-IV and ERIC-V types, each contains eight isolates.

### Results of antibiotic susceptibility testing

**Fig. 3:**
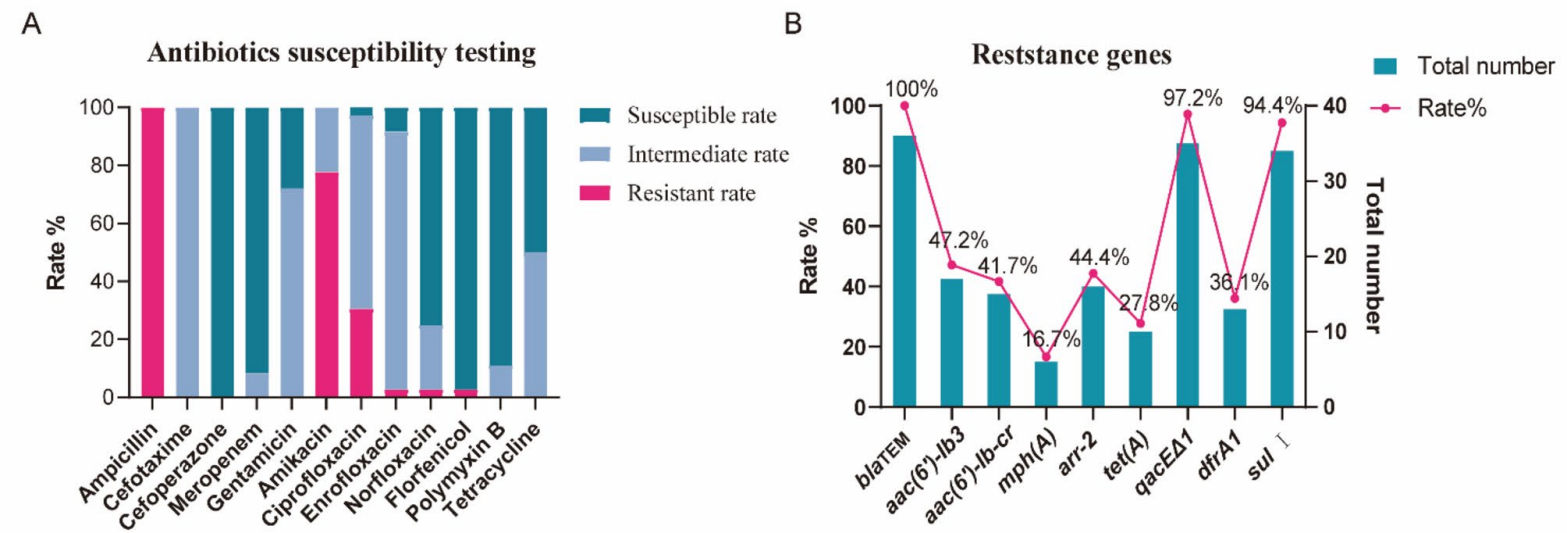
Antibiotic susceptibility testing and detection of antibiotic resistance genes of all the 36 strains of *P. shigelloides* isolated from crested ibis. Percentage distribution of antibiotics susceptibility testing (**A**) and percentage of antibiotic resistance genes (**B**) of all the 36 *P. shigelloides* strains isolated from crested ibis

In order to further understand the antibiotic resistance patterns of these isolates, susceptibility of all isolates to different antibiotics were determined using the disk diffusion method on Mueller-Hilton agar plates according to recommendations of CLSI. The results show that the highest prevalence of resistance was observed for ampicillin (36/36 = 100%), followed by amikacin (28/36 = 77.8%) and ciprofloxacin (11/36 = 30.6%). Enrofloxacin, norfloxacin and florfenicol resistance was only detected in one isolate (*P. shigelloides-*21, *P. shigelloides-*29 and *P. shigelloides-*33, respectively) (Fig. 3A, Table S2). Specifically, 27.8% (10/36) of the isolates were multidrug resistant (resistant to three or more categories of antimicrobials) (Table S2).

### Genome sequencing and phylogenetic analysis of the representative strains

According to the results of molecular typing and drug resistance, ten representative strains were selected for next-generation sequencing. The genome assembly results showed that the number of raw reads produced by ten representative strains of *P. shigelloides* ranged from 8,943,568 to 15,067,496 and the genome length were ranged from 3,614,060 bp to 3,809,779 bp, and the average content of GC was 51.8% (ranged from 51.59 to 51.95%) (Table 1).

**Table 1:** Genome assembly results of 10 representative strains of *P. shigelloides*

Sample name	Raw reads	Length (bp)	GC%	Scaffolds count	GenBank accession
*P. shigelloides*-3	15,067,496	3,614,076	52.0%	38	JBJKNT000000000
*P. shigelloides*-6	9,280,266	3,631,606	51.9%	44	JBJKNU000000000
*P. shigelloides*-12	8,943,568	3,630,500	51.9%	44	JBJKNV000000000
*P. shigelloides*-15	9,501,882	3,778,495	51.6%	50	JBJKNW000000000
*P. shigelloides*-18	10,063,078	3,754,058	51.6%	63	JBJKNX000000000
*P. shigelloides*-20	12,691,512	3,619,221	51.9%	40	JBJKNY000000000
*P. shigelloides*-25	10,168,198	3,614,060	52.0%	40	JBJKNZ000000000
*P. shigelloides*-29	10,631,404	3,631,315	51.9%	45	JBJKOA000000000
*P. shigelloides*-32	13,700,446	3,631,380	51.9%	44	JBJKOB000000000
*P. shigelloides*-36	8,866,396	3,809,779	51.8%	35	JBJKOC000000000

**Fig. 4:**
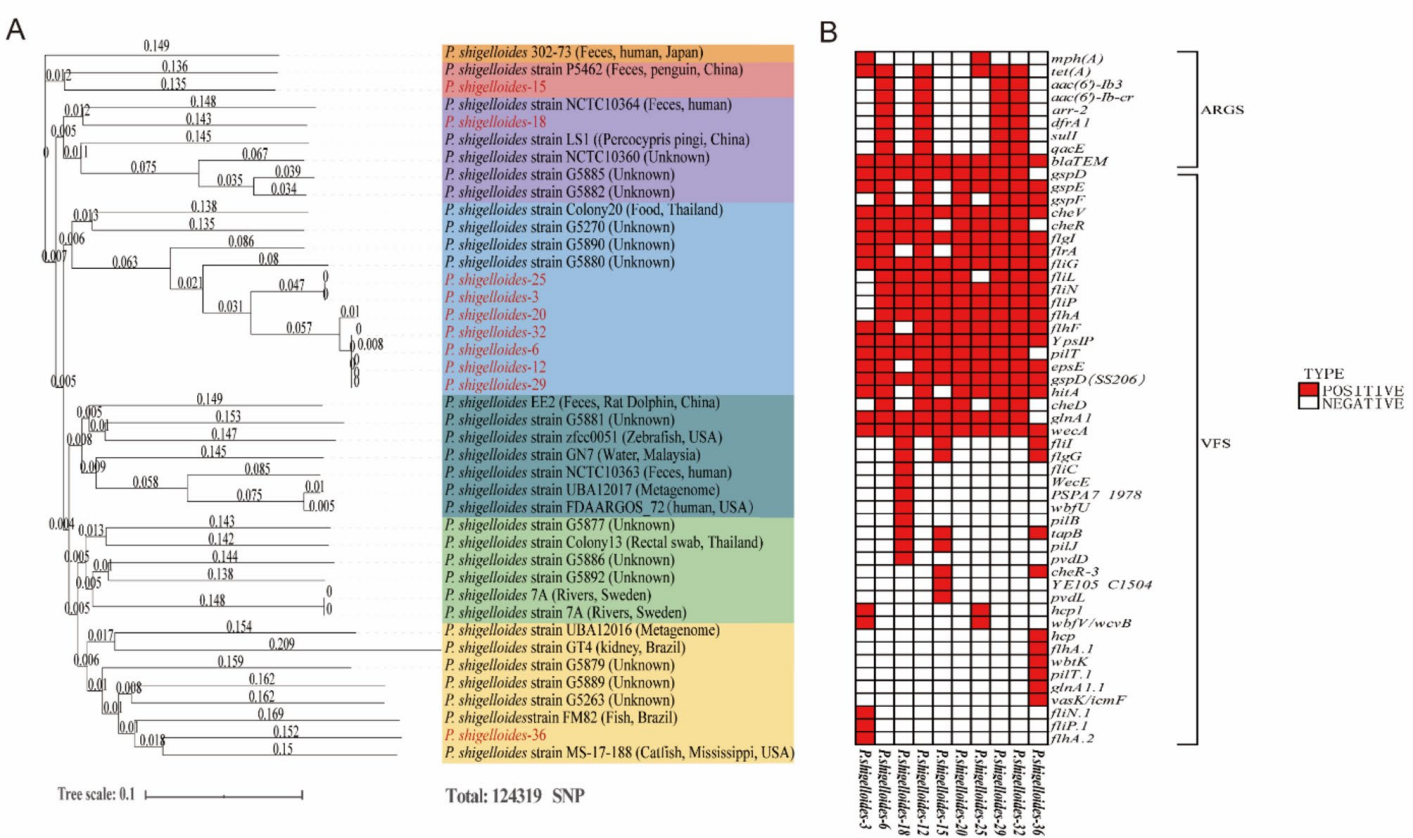
Phylogenetic tree based on the genomes of *P. shigelloides* and analysis of virulence related and antibiotic resistance genes. (**A**) Ten representative strains were selected for next-generation sequencing and the phylogenomic analysis of *P. shigelloides* basing on SNP (Parsnp) were performed. The isolates in this study were labeled in red. (**B**) Whole-genome sequences were compared with the Virulence Factor Database (VFDB) and the Comprehensive Antibiotic Research Database (CARD) respectively. The distribution of virulence genes and drug resistance genes of the ten isolates are represented by a heat map. The positives were labeled in red and the negatives were represented by blanks

Subsequently, the phylogenomic analysis of *P. shigelloides* based on SNP (Parsnp) was performed, which contained ten representative strains in this study and thirty-one strains downloaded from PATRIC database (Table S1). The results showed that the sequences of these strains exhibited a quite distinct difference in overall similarity, yet it is still can be seen that the ten representative strains possibly originated from different sources (Fig. 4A). 70.0% (7/10) of the representative strains of *P. shigelloides* isolated from the crested ibis belong to a distinct phylogenetic branch that does not include strains from other sources. These strains are clustered with a foodborne isolate, *P. shigelloides* strain Colony20 from Thailand, indicating a degree of species specificity among certain strains of *P. shigelloides*, including those from the crested ibis. In contrast, the remaining three *P. shigelloides* isolates in this study are associated with different sources. Specifically, *P. shigelloides*-15 is clustered with *P. shigelloides* strain P5462, isolated from a penguin in Hong Kong. *P. shigelloides*-36 is clustered with *P. shigelloides* strain MS-17-188, derived from a catfish in eastern Mississippi, USA. Notably, *P. shigelloides*-18 is clustered with *P. shigelloides* strain NCTC10364, isolated from human feces in Japan.

### Analysis of virulence related and antibiotic resistance genes in sequenced genomes

Furthermore, the virulence-related and antibiotic resistance genes of these stains were analyzed. Whole-genome sequences were submitted and compared using the VFDB and CARD platforms, respectively. The results revealed that a total of 45 virulence genes were predicted in ten representative strains of *P. shigelloides*, including 16 core virulence genes. All the strains carried genes of flagellar P-ring protein (*flgI*), flagellar switch protein (*fliG*), general secretory pathway protein D (*gspD SS206*), capsular polysaccharide (*wecA*) and double-component bacterial regulation (*cheV*). There were nine strains carried the genes of flagellar switch protein (*fliN*) and general secretory pathway protein E (*epsE*), and eight strains carried sigma-54-dependent transcriptional activator (*flrA*). All representative strains in this study lacked genes related to the cytotoxic outer membrane protein ComP. Notably, all strains harbored over twenty virulence genes, with *P. shigelloides-*18 and *P. shigelloides-*36 possessing the highest number of virulence genes, at twenty-four and twenty-six, respectively (Fig. 4B). Notably, some unique virulence genes, such as *hcp*, *flhA.1*, *wbtK*, *pilT.1*, *glnA1.1* and *vasK*/*icmF* only distributed in *P. shigelloides*-36.

Meanwhile, nine common antibiotic resistant genes were detected in the ten strains of *P. shigelloides* as well, including *bla*_TEM_, *mph(A)*, *tet(A)*, *arr-2*, *aac(6’)-Ib-cr*, *aac(6’)-Ib3*, *dfrA1*, *sulI* and *qacEΔ1*, which were related to β-lactams, macrolides, tetracycline, rifamycin, quinolones, aminoglycosides, folic acid antagonist, sulfonamides and quaternary ammonium compounds resistance, respectively. Among them, *bla*_TEM_ was distributed in all the ten strains (100%) and *tet(A)* was detected in six strains (60.0%). *Arr-2*,* aac(6’)-Ib-cr*,* aac(6’)-Ib3*,* dfrA1*,* sulI* and *qacEΔ1* were positive in four strains (40.0%). *Mph(A)* was distributed in two strains (20.0%). Six strains were identified as carrying more than three antibiotic-resistant genes, among which four strains carried eight resistance genes (*P. shigelloides*-6, *P. shigelloides-*12, *P. shigelloides-*29, *P. shigelloides-*32) (Fig. 4B).

Furthermore, based on the results of whole genome sequencing, the above nine resistance genes were detected by PCR and sequencing for the remaining *P. shigelloides* strains. The prevalence of antibiotic resistance genes was shown in Fig. 3B. The prevalence of the *bla*_TEM_ gene is the highest at 100%, followed by *qacEΔ1* at 97.2% and *sulI* at 94.4%. The lowest positive rate is observed for *mph(A)* at 16.7%, while the carrying rates for other resistance genes range from 27.8 to 47.2%. Overall, 97.2% of the strains tested positive for more than two resistance genes.

### Pathogenicity of the isolates in mice and ducks

**Fig. 5:**
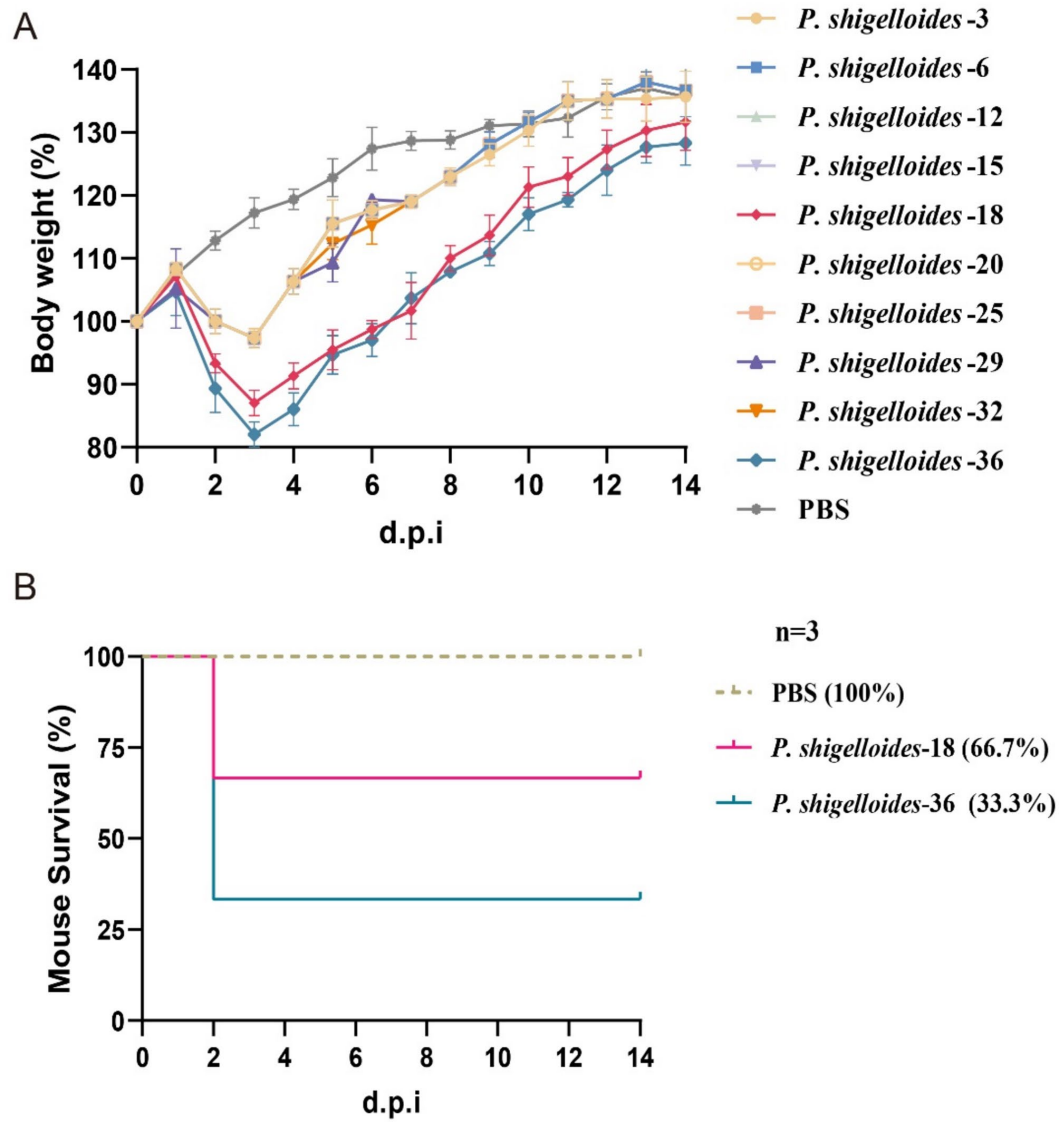
**Pathogenicity of the*P. shigelloides*isolates in mice.** The weight loss of mice infected by the ten representative *P. shigelloides* (A) and survival rate of mice infected by two strains (*P. shigelloides*-18 and *P. shigelloides* 36) (B) were monitored for 14 d.p.i. Data are presented as mean ± standard deviations (SDs)

The two tested strains (*P. shigelloides*-18 and *P. shigelloides*-36) showed pathogenicity to both mice and ducks. Ducks exhibited diarrhea on the second day after challenging, but none of the ducklings died (data not shown). Mice infected with *P. shigelloides*-18 and *P. shigelloides*-36 exhibited significant weight loss, amounting to 15% and 20%, respectively, by the third day post-infection (Fig. 5A). Notably, *P. shigelloides*-18 and *P. shigelloides*-36 resulted in case fatality rates of 33.3% and 66.7% in mice by the second day post-infection (Fig. 5B). Additionally, swelling of the spleens was observed in mice, from which *P. shigelloides* was subsequently recovered (data not shown).

## Discussion

Wild birds have received widespread attention as reservoirs for various pathogens, especially zoonotic pathogens [25, 26]. As a rare wild bird in China, the crested ibis has been found to be infected with *E. coli*, *Chlamydia psittaci*, *Chlamydia ibidis*, and Newcastle disease viruses previously [27–29]. *P. shigelloides* is an important conditional and potential pathogen for humans and animals, which could be isolated from the environment and various animals [6, 30]. In this study, *P. shigelloides* was the first time isolated from crested ibis’ population with a high positivity rate (about 45.6%). The genetic evolution analysis conducted in this study revealed that the *P. shigelloides* isolates found in the crested ibis population possibly originated from diverse sources, including humans, penguins, catfish, and various environmental settings (Fig. 4A). This finding underscores the potential for cross-regional and cross-species transmission of *P. shigelloides*, although the specific transmission pathways warrant further investigation. Additionally, the antibiotic resistance and virulence profiles of these *P. shigelloides* strains have garnered increased attention.

Antibiotic resistance has long been a threat to global public health and sustainable development, and this major scientific issue involves multiple fields [31, 32]. *P. shigelloides* are potential pathogens associated with intestinal diseases, and most gastrointestinal diseases caused by this bacterium do not require medical intervention [33]. However, some moderate to severe diarrhea cases often necessitate appropriate antibiotic treatment [33]. In a study of antibiotic susceptibility testing for 54 *P. shigelloides* strains causing diarrhea, 22.2% (12/54) of the strains were multidrug resistant, with resistance to ampicillin, tetracycline, erythromycin being 77.7%, 29.2%, and 18.5%, respectively [34]. *P. shigelloides* isolated from the endangered Chinese sturgeon (*Acipenser sinensis*) demonstrated resistance to ampicillin, penicillin, midecamycin, oxacillin, and clindamycin, while exhibiting sensitivity to cefatriaxone, piperacillin, cefoperazone, cefazolin, and ciprofloxacin [9]. Additionally, Selim et al. confirmed that *P. shigelloides* isolated from shellfish and aquatic samples displayed varying degrees of susceptibility to the tested antimicrobial agents, including nalidixic acid, carbenicillin, cephalothin, erythromycin, kanamycin, tetracycline, and ciprofloxacin, with kanamycin and tetracycline proving to be the most effective antibiotics against *P. shigelloides*, achieving effectiveness rates of 87% [35].

In this study, the results of antibiotic susceptibility testing and resistance genes identification show that all *P. shigelloides* bacteria are resistant to ampicillin and carry the *bla*_TEM_ gene (Table S2, Fig. 3A and B). Since the breeding base for the crested ibis has never used ampicillin, these strains may be naturally resistant to ampicillin. Avison et al. reported that some *P. shigelloides* are naturally resistant to ampicillin, which is consistent with our findings [36]. Though a comprehensive study on *Plesiomonas* diarrhea revealed that 85% of the cases resolved on their own, antimicrobial treatment might be required for moderate to severe diarrhea [37]. Ciprofloxacin is one of the recommended antibiotics for treating severe dysentery-like diarrhea caused by *P. shigelloides* [38]. Meanwhile, aminoglycoside antibiotics, particularly amikacin, reduced the surface hydrophobicity and motility of *P. shigelloides* strains, making it a promising treatment option [39]. In this study, certain strains showed different levels of resistance to amikacin and ciprofloxacin, primarily due to the resistance genes *aac(6’)-Ib3* and *aac(6’)-Ib-cr*. *aac(6’)-Ib3* belong to the *aac(6’)* gene family coding for an aminoglycoside acetyltransferase AAC(6’) that acetylates the 6’-amino group of 4,6-disubstituted 2-deoxystreptamines such as amikacin [40]. AAC(6’)-Ib-cr, a variant of the gene for aminoglycoside acetyltransferase AAC(6’)-Ib, diminishes ciprofloxacin’s effectiveness by N-acetylating the amino nitrogen on its piperazinyl group [41]. Being one of the plasmid mediated quinolone resistance (PMQR) genes, *aac(6’)-Ib-cr* is frequently found in gram-negative bacteria resistant to fluroquinolones [42, 43]. Also, the possibility by the overexpression of certain efflux pumps cannot be dismissed.

Fortunately, only 27.8% of the strains exhibited multidrug resistance, with over 90.0% of the strains remaining sensitive to cefoperazone, florfenicol, and meropenem. This suggests that the antibiotic resistance of *P. shigelloides* in the crested ibis’ population is not severe, possibly due to the fact that crested ibis breeding centers hardly use antibiotics and strictly follows the feed hygiene standards of GB 2707, GB 2733, and GB 13,078 (Chinese national standard) for formulating crested ibis’ feed. Despite the fact that these crested ibis have rarely been given antibiotics, the isolated *P. shigelloides* still possess multiple antibiotic resistance genes. Given that *P. shigelloides* are quite often detected in water habitats and on aquatic creatures like fish, shellfish, and certain waterfowls, the *P. shigelloides* identified in crested ibis likely originates from these sources [8–10, 44]. Consequently, the antibiotic resistance genes found in these *P. shigelloides* isolates could be inherent [45] or acquired from commensal bacteria in aquatic environments through plasmids or horizontal gene transfer [46, 47]. As a prevalent pathogen among aquatic animals, the presence of resistance genes in *P. shigelloides* poses a potential threat to global public health from a “One Health” perspective [48].

The infection of *P. shigelloides* is complex, and many virulence genes are involved, such as hemolysin, flagellin, elastase, enterotoxin, cholera-like toxins, cytotoxic outer membrane protein (*comP*), and the factors related to secretion system [35, 49, 50]. Among these, the cytotoxic outer membrane protein ComP may be a major virulence factor responsible for host mortality after infection [49], while hemolysins, enterotoxins, and cholera-like toxins can cause varying degrees of damage to the host [51]. Fimbriae, capsules, and secretory system-related proteins play a significant role in promoting bacterial growth and enhancing their competitive advantages [35, 50].

In this study, mice and Muscovy ducklings were utilized as model organisms to assess the pathogenicity of representative isolates of *P. shigelloides* isolates. Pathogenicity assessments have demonstrated that *P. shigelloides* isolates exhibit relatively low pathogenic potential, which may be attributed to several factors. Firstly, the representative isolates employed in this study were deficient in the critical virulence factor, the cytotoxic outer membrane protein ComP. Additionally, given that these isolates were derived from the crested ibis, they may show reduced susceptibility to causing infections in Muscovy ducklings and mice. Due to the endangered status of the crested ibis, utilizing this species for pathogenicity assessments is not feasible. Nonetheless, it is important to note that the isolates have been observed to cause mortality in mice. Especially, *P. shigelloides*-18 and *P. shigelloides*-36 appeared to exhibit greater pathogenicity in mouse models compared to other strains. The explanation might be that *P. shigelloides*-18 and *P. shigelloides*-36 contain the highest counts of virulence genes, numbering twenty-four and twenty-six, respectively (Fig. 4B). Notably, distinct virulence genes, including *hcp*, *flhA.1*, *wbtK*, *pilT.1*, *glnA1.1*, and *vasK*/*icmF*, are exclusively found in *P. shigelloides*-36, while *fliC*, *wecE*, *PSPA7*, *wbfU*, and *pliB* are unique to *P. shigelloides*-18 (Fig. 4B). These virulence factors play a role in flagellum formation, hemolysin regulation, adherence and attachment to villi, carbohydrate metabolism, and more, potentially enhancing the strains’ virulence [35, 38, 52, 53]. Future research should prioritize enhancing health monitoring protocols for the crested ibis and evaluating the impact of *P. shigelloides* on the health of this species.

In summary, for the first time, we have isolated *P. shigelloides* from the endangered crested ibis (36/79, 45.6%). These *P. shigelloides* exhibited varying degrees of antibiotic resistance, with a multidrug resistance rate of 27.8%, and carried different numbers of virulence genes. The pathogenicity observed in mice and Muscovy ducks indicates that the representative *P. shigelloides* strains had relatively weak pathogenicity, but further investigation is still needed. Whole genomic analysis shows that *P. shigelloides* strains from the crested ibis exhibit phylogenetic relationships with *P. shigelloides* from various sources, including penguins, catfishes, environments, and even humans. This suggests that *P. shigelloides* from the crested ibis may disseminate across various environments and pose a potential health risk to humans. This highlights the necessity for increased surveillance of the bacteria from the crested ibis and its habitat to safeguard the health of both animals and humans.

## Electronic supplementary material

Below is the link to the electronic supplementary material.

Supplementary Material 1

## Data Availability

Data is provided within the manuscript or supplementary files. Sequence data that support the findings of this study have been deposited in the GenBank with the primary accession number JBJKNT000000000, JBJKNU000000000, JBJKNV000000000, JBJKNW000000000, JBJKNX000000000, JBJKNY000000000, JBJKNZ000000000, JBJKOA000000000, JBJKOB000000000, and JBJKOC000000000, which are also stated in the manuscript. Uncropped gel image for ERIC-PCR of Figure 2B is presented in Figure S1 in supplementary files.
