# Supplementary material for: First report of multidrug-resistant and pathogenic Plesiomonas shigelloides from endangered crested ibis (Nipponia nippon)
Source: BMC Vet Res. 2025 Apr 29;21:299. doi: 10.1186/s12917-025-04755-3 (PMC12038971; doi:10.1186/s12917-025-04755-3)
Supplement: Supplementary file 1 — Supplementary Material 1 [file 12917_2025_4755_MOESM1_ESM.docx]

**Table S1.** Genome information of *P. shigelloides* strains performed for the phylogenomic analysis in Figure 3A

| **Genome name** | **Source** | **CDS** | **GC content/%** | **Length** | **ID** |
| --- | --- | --- | --- | --- | --- |
| *P. shigelloides* 302-73 | Feces, human, Japan | 3277 | 51.2 | 3908112 | 1315976.3 |
| *P. shigelloides* strain FM82 | Fish, Brazil | 3589 | 51.54 | 3726260 | 703.23 |
| *P. shigelloides* strain MS-17-188 | Catfish, Eastern Mississippi, USA | 3724 | 51.45 | 3970359 | 703.24 |
| *P. shigelloides* EE2 | Feces, Rat Dolphin, China | 3366 | 51.87 | 3660999 | 703.31 |
| *P. shigelloides* strain UBA12016 | Metagenome | 3093 | 52.87 | 2998852 | 703.32 |
| *P. shigelloides* strain UBA12017 | Metagenome | 3496 | 51.64 | 2859263 | 703.33 |
| *P. shigelloides* strain NCTC10363 | Feces, human | 3481 | 51.65 | 3839096 | 703.34 |
| *P. shigelloides* strain NCTC10364 | Feces, human | 3293 | 51.92 | 3653255 | 703.45 |
| *P. shigelloides* strain G5877 | Unknown | 3517 | 51.66 | 3760261 | 703.58 |
| *P. shigelloides* strain G5879 | Unknown | 3584 | 51.52 | 3778053 | 703.52 |
| *P. shigelloides* strain G5880 | Unknown | 3295 | 51.97 | 3557298 | 703.55 |
| *P. shigelloides* strain G5881 | Unknown | 3604 | 51.64 | 3788257 | 703.53 |
| *P. shigelloides* strain G5882 | Unknown | 3525 | 51.63 | 3783663 | 703.48 |
| *P. shigelloides* strain G5885 | Unknown | 3494 | 51.77 | 3761499 | 703.50 |
| *P. shigelloides* strain G5886 | Unknown | 3448 | 51.8 | 3689697 | 703.54 |
| *P. shigelloides* strain G5889 | Unknown | 3619 | 51.53 | 3763376 | 703.51 |
| *P. shigelloides* strain G5890 | Unknown | 3336 | 51.93 | 3566572 | 703.49 |
| *P. shigelloides* strain G5892 | Unknown | 3367 | 51.96 | 3668125 | 703.56 |
| *P. shigelloides* strain G5270 | Unknown | 3833 | 51.3 | 3960408 | 703.57 |
| *P. shigelloides* strain G5263 | Unknown | 3351 | 51.78 | 3604750 | 703.59 |
| *P. shigelloides* strain FDAARGOS_725 | Clinical isolates, human, USA | 3504 | 51.62 | 3827531 | 703.60 |
| *P. shigelloides* GN7 | Water, Malaysia | 3380 | 51.61 | 3916235 | 703.7 |
| *P. shigelloides* strain NCTC10360 | Unknown | 3037 | 52.03 | 3405979 | 703.8 |
| *P. shigelloides* strain P5462 | Feces, Penguins, Hong Kong | 3521 | 51.8 | 3843104 | 703.80 |
| *P. shigelloides* strain zfcc0051 | Zebrafish, USA | 4233 | 51.74 | 4037587 | 703.85 |
| *P. shigelloides* strain Colony20 | Food, Thailand | 2937 | 52.61 | 3406079 | 703.86 |
| *P. shigelloides* strain Colony13 | Rectal swab, Thailand | 2943 | 52.6 | 3406079 | 703.87 |
| *P. shigelloides* strain GT4 | Kidney, Brazil | 3375 | 51.95 | 3673619 | 703.89 |
| *P. shigelloides* strain LS1 | Percocypris pingi, China | 3605 | 51.61 | 3866061 | 703.9 |
| *P. shigelloides* strain 7A | Rivers, Sweden | 3525 | 51.72 | 3860786 | 703.94 |
| *P. shigelloides* 7A | Rivers, Sweden | 3525 | 51.72 | 3860786 | 703.95 |
| *P. shigelloides*-6 | Crested Ibis, Deqing County, Zhejiang Province, China | 3103 | 51.85 | 3631606 | This study |
| *P. shigelloides*-18 | Crested Ibis, Deqing County, Zhejiang Province, China | 3121 | 51.82 | 3754058 | This study |
| *P. shigelloides*-12 | Crested Ibis, Deqing County, Zhejiang Province, China | 3263 | 52.06 | 3630500 | This study |
| *P. shigelloides*-15 | Crested Ibis, Deqing County, Zhejiang Province, China | 3120 | 51.36 | 3778495 | This study |
| *P. shigelloides*-20 | Crested Ibis, Deqing County, Zhejiang Province, China | 3256 | 51.95 | 3619221 | This study |
| *P. shigelloides*-25 | Crested Ibis, Deqing County, Zhejiang Province, China | 3131 | 52.04 | 3614060 | This study |
| *P. shigelloides*-29 | Crested Ibis, Deqing County, Zhejiang Province, China | 3104 | 51.98 | 3631315 | This study |
| *P. shigelloides*-32 | Crested Ibis, Deqing County, Zhejiang Province, China | 3118 | 51.95 | 3631380 | This study |
| *P. shigelloides*-36 | Crested Ibis, Deqing County, Zhejiang Province, China | 3119 | 51.63 | 3809779 | This study |
| *P. shigelloides*-3 | Crested Ibis, Deqing County, Zhejiang Province, China | 3354 | 52.02 | 3614076 | This study |

**Table S2.** Detection of antibiotics resistance patterns of all the 36 strains of *P. shigelloides* isolated from crested ibis^#^

| **Strains** | **Tested antibiotics** | | | | | | | | | | | |
| --- | --- | --- | --- | --- | --- | --- | --- | --- | --- | --- | --- | --- |
|  | **AMP** | **CTX** | **CPZ** | **MEM** | **GM** | **AMK** | **CIP** | **ENR** | **NOR** | **FFC** | **PMB** | **TET** |
| ***P. Shigelloides*-1** | **R** | **I** | **S** | **S** | **I** | **R** | **R** | **I** | **S** | **S** | **S** | **I** |
| ***P. Shigelloides*-2** | **R** | **I** | **S** | **S** | **I** | **R** | **I** | **I** | **S** | **S** | **S** | **S** |
| ***P. Shigelloides*-3** | **R** | **I** | **S** | **S** | **I** | **I** | **I** | **I** | **S** | **S** | **S** | **I** |
| ***P. Shigelloides*-4** | **R** | **I** | **S** | **S** | **S** | **R** | **I** | **I** | **S** | **S** | **S** | **I** |
| ***P. Shigelloides-*5** | **R** | **I** | **S** | **S** | **I** | **R** | **I** | **I** | **S** | **S** | **S** | **S** |
| ***P. Shigelloides-*6** | **R** | **I** | **S** | **S** | **I** | **R** | **R** | **I** | **S** | **S** | **S** | **I** |
| ***P. Shigelloides-*7** | **R** | **I** | **S** | **S** | **I** | **R** | **R** | **I** | **I** | **S** | **I** | **I** |
| ***P. Shigelloides-*8** | **R** | **I** | **S** | **S** | **S** | **R** | **I** | **I** | **I** | **S** | **S** | **S** |
| ***P. Shigelloides-*9** | **R** | **I** | **S** | **S** | **S** | **I** | **S** | **S** | **S** | **S** | **S** | **S** |
| ***P. Shigelloides*-10** | **R** | **I** | **S** | **S** | **S** | **R** | **R** | **I** | **S** | **S** | **S** | **S** |
| ***P. Shigelloides-*11** | **R** | **I** | **S** | **S** | **I** | **R** | **I** | **I** | **S** | **S** | **S** | **S** |
| ***P. Shigelloides*-12** | **R** | **I** | **S** | **S** | **I** | **R** | **R** | **I** | **S** | **S** | **S** | **I** |
| ***P. Shigelloides*-13** | **R** | **I** | **S** | **S** | **S** | **I** | **I** | **I** | **S** | **S** | **S** | **I** |
| ***P. Shigelloides*-14** | **R** | **I** | **S** | **S** | **I** | **R** | **R** | **I** | **I** | **S** | **S** | **I** |
| ***P. Shigelloides*-15** | **R** | **I** | **S** | **S** | **S** | **I** | **R** | **I** | **S** | **S** | **S** | **S** |
| ***P. Shigelloides*-16** | **R** | **I** | **S** | **S** | **S** | **R** | **I** | **I** | **I** | **S** | **S** | **S** |
| ***P. Shigelloides*-17** | **R** | **I** | **S** | **S** | **S** | **R** | **I** | **I** | **S** | **S** | **S** | **S** |
| ***P. Shigelloides*-18** | **R** | **I** | **S** | **S** | **I** | **R** | **I** | **I** | **I** | **S** | **S** | **S** |
| ***P. Shigelloides*-19** | **R** | **I** | **S** | **S** | **I** | **R** | **I** | **I** | **S** | **S** | **I** | **I** |
| ***P. Shigelloides*-20** | **R** | **I** | **S** | **I** | **I** | **R** | **I** | **I** | **S** | **S** | **S** | **S** |
| ***P. Shigelloides*-21** | **R** | **I** | **S** | **I** | **I** | **R** | **I** | **R** | **I** | **S** | **S** | **I** |
| ***P. Shigelloides*-22** | **R** | **I** | **S** | **S** | **I** | **R** | **I** | **I** | **S** | **S** | **S** | **S** |
| ***P. Shigelloides*-23** | **R** | **I** | **S** | **S** | **I** | **R** | **I** | **I** | **S** | **S** | **S** | **S** |
| ***P. Shigelloides*-24** | **R** | **I** | **S** | **S** | **I** | **I** | **I** | **I** | **S** | **S** | **S** | **I** |
| ***P. Shigelloides*-25** | **R** | **I** | **S** | **S** | **I** | **I** | **R** | **I** | **S** | **S** | **I** | **I** |
| ***P. Shigelloides*-26** | **R** | **I** | **S** | **S** | **I** | **R** | **I** | **S** | **S** | **S** | **S** | **S** |
| ***P. Shigelloides*-27** | **R** | **I** | **S** | **S** | **I** | **R** | **I** | **I** | **S** | **S** | **S** | **S** |
| ***P. Shigelloides*-28** | **R** | **I** | **S** | **S** | **I** | **R** | **R** | **I** | **S** | **S** | **S** | **I** |
| ***P. Shigelloides*-29** | **R** | **I** | **S** | **S** | **I** | **I** | **I** | **I** | **R** | **S** | **S** | **I** |
| ***P. Shigelloides*-30** | **R** | **I** | **S** | **S** | **I** | **R** | **I** | **I** | **S** | **S** | **S** | **I** |
| ***P. Shigelloides*-31** | **R** | **I** | **S** | **S** | **I** | **R** | **R** | **I** | **S** | **S** | **S** | **I** |
| ***P. Shigelloides*-32** | **R** | **I** | **S** | **S** | **I** | **R** | **R** | **I** | **S** | **S** | **I** | **I** |
| ***P. Shigelloides*-33** | **R** | **I** | **S** | **S** | **S** | **I** | **I** | **I** | **I** | **R** | **S** | **S** |
| ***P. Shigelloides*-34** | **R** | **I** | **S** | **S** | **I** | **R** | **I** | **I** | **S** | **S** | **S** | **I** |
| ***P. Shigelloides*-35** | **R** | **I** | **S** | **I** | **I** | **R** | **I** | **I** | **I** | **S** | **S** | **S** |
| ***P. Shigelloides*-36** | **R** | **I** | **S** | **S** | **S** | **R** | **I** | **S** | **S** | **S** | **S** | **S** |

^#^Susceptibility was categorized as susceptible or resistant by measuring the diameter of inhibition zone according to the criteria stipulated by the CLSI. “R”, resistance; “I”, intermediate; “S”, susceptible.

AMP, ampicillin; CTX, cefotaxime; CPZ, cefoperazone; MEM, meropenem; GM, gentamicin; AMK, amikacin; CIP, ciprofloxacin; ENR, enrofloxacin; NOR, norfloxacin; FFC, florfenicol; PMB, polymyxin B; TET, tetracycline.

**
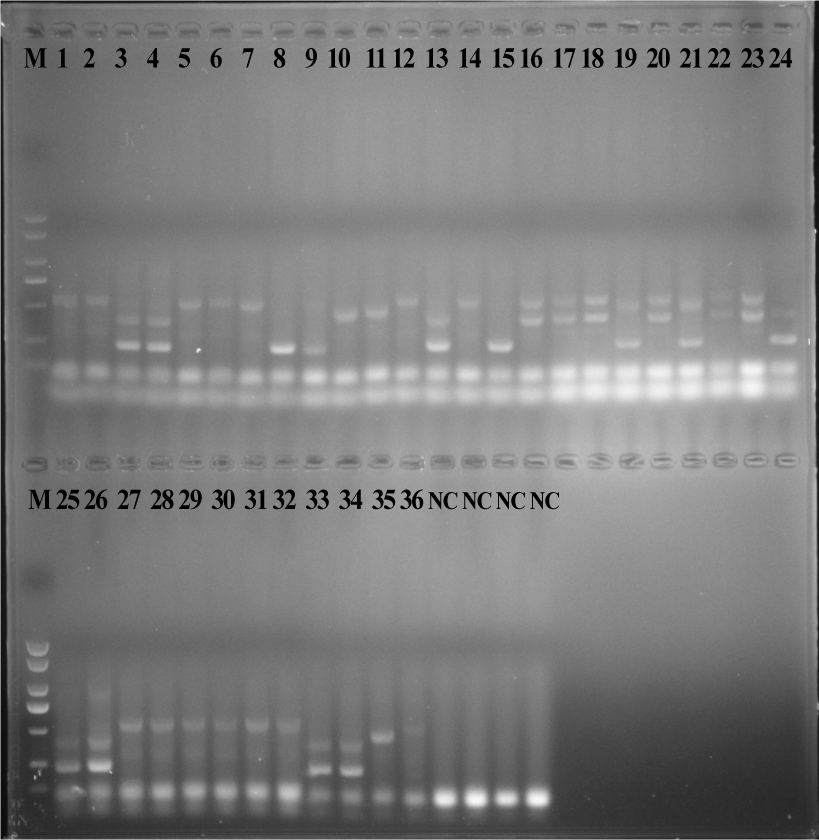
**

**Figure S1.** Uncropped gel image for ERIC-PCR of Figure 2B. 1-36, *P. shigelloides* isolates 1-36; M, DL2000 bp DNA marker; NC, negative control.
